# Efficacy of Clear Aligners in Treating Class III Malocclusion With Mandibular Molar Distalization: A Systematic Review

**DOI:** 10.7759/cureus.48134

**Published:** 2023-11-01

**Authors:** Aishwarrya Padmanabhan, Yusuf Khan, Vikrant Lambate, Ushanandhini K, Niha Naveed, Mansi Singh, Puneet Kamal Nagi

**Affiliations:** 1 Orthodontics and Dentofacial Orthopedics, Sri Ramakrishna Dental College and Hospital, Coimbatore, IND; 2 Orthodontics and Dentofacial Orthopedics, Diamond Medical Specialists, Taif, SAU; 3 Orthodontics and Dentofacial Orthopedics, Mahatma Gandhi Missions (MGM) Dental College and Hospital, Navi Mumbai, IND; 4 Orthodontics and Dentofacial Orthopedics, Saveetha Dental College, Chennai, IND; 5 Orthodontics and Dentofacial Orthopedics, Kalka Dental College and Hospital, Meerut, IND; 6 Periodontics, Punjab Government Dental College and Hospital, Amritsar, IND

**Keywords:** third molar, mandibular prognathism, facial profile, clear aligners, orthodontic therapy, mandibular molar distalization, class iii malocclusion

## Abstract

The primary goal of orthodontic therapy in pseudo-class III is to restore the proper dental connection by rectifying the canine and molar relationship to Class I through lower molar and premolar visualization, as well as providing normal anterior overjet. The purpose of this systematic study was to determine the efficacy of clear aligners in treating class III malocclusion with mandibular molar distalization. A wide range of searches were done on various search engines like Cochrane, Web of Science, Embase, PubMed, Scopus, and Google Scholar to collect relevant articles related to our study. This review's article selection was guided by the PRISMA flowchart. The electronic findings provided numerous articles with nearly 78 articles regarding clear aligners in class III malocclusion with molar distalization. From this, seven full-text papers were evaluated for eligibility criteria, with two articles being rejected with justification and five articles being elaborated in the current systematic review. The current evidence of this review suggested that the clear aligners were effective in correcting class III malocclusion with molar distalization. The amount of molar distalization is about 2 to 3 mm, which helps in achieving molar and canine relationship in class I, with a high compliance level and also improvement of the facial profile.

## Introduction and background

Class III malocclusion is a dentofacial clinical condition characterized by a concave profile produced by a forwardly positioned mandible, a backwardly positioned maxilla, or a combination of both [1]. It is critical to distinguish between basal pseudo class III and skeletal class III malocclusions in non-growing individuals. Skeletal basal class III is caused by a deformity in the maxilla or mandible, which necessitates surgical treatment, whereas functional or pseudo-class III is caused by a discrepancy between maximal intercuspation and centric occlusion, resulting in occlusal disturbance in which the patient moves the mandible forward while closing the jaw for maximal intercuspation. Orthodontic therapy alone can address this occlusal disruption [2,3].

The primary goal of orthodontic therapy in pseudo-class III is to achieve proper dental relations by rectifying canine and molar relationships to class I malocclusion through distalization of lower premolar and molar, as well as providing normal anterior overjet [4,5]. Lower molar distalization is a time-consuming procedure due to its higher bone density and radicular morphology [6]. Various techniques are employed, such as class III elastics, open coil springs, fixed appliance therapy, functional appliances like lip bumpers and removable appliances with distal expansion screws [7].

In recent times, most adult patients have been in the working environment and so they ask for less visible and more comfortable orthodontic treatment which led to a new field of clear aligner therapy. With better treatment regimens and patient compliance, they are a better option for fixed multibracket therapy. Clear aligner therapy produces clinical results comparable to fixed orthodontic treatment [8,9]. Patients treated with clear aligners have good periodontal health and fewer white spot lesions during the treatment. They can be used in mild to moderate crowding cases but caution must be exercised in complex cases. The chair time is found to be significantly shorter in the clear aligners group, allowing the clinician to treat more patients as compared to fixed appliance therapies [9].

There has been little study on the effectiveness of clear aligners in distalizing upper molars. As a result, the present systematic review was conducted to assess the effectiveness of clear aligners in treating class III malocclusion with mandibular molar distalization.

## Review

Methods

Materials and Methods

The current review was written in accordance with the PRISMA guidelines [10]. The study technique for our systematic review was based on the principles of the Cochrane Handbook for Systematic Reviews of Interventions [11].

Study Question

The targeted question was “How effective can the clear aligners treat class III malocclusion with mandibular molar distalization?”

Search Strategy

In this review, we have made a cross-disciplinary search and included retrospective studies and case series regarding the efficacy of clear aligners in treating class III malocclusion with mandibular molar distalization. The study information was obtained from the original articles, systematic reviews, and relevant citations and bibliographies with the search topic of the efficacy of clear aligners in treating class III malocclusion. A wide range of searches was done on various search engines such as Cochrane, Web of Science, Embase, PubMed, Scopus, and Google Scholar to collect relevant articles related to our study. Articles published from January 2000 to July 2023 were checked without excluding any article on its published year basis or language basis.

For PubMed, the MeSH words used were “clear aligners”, “Invisalign”, “lower molar distalization”, and “class III malocclusion” with Boolean operators “AND”, and “OR” to refine the search were used for the search strategy. In Embase, the search strategy was done with Emtree keywords in the pattern as follows: (“Clear aligners”/exp OR “CAT”/exp OR Invisalign) AND (“lower molar distalization”/exp OR “mandibular molar distalisation”/exp OR “lower first molar distalization”) AND (“class III malocclusion”/exp OR “dental class III occlusion”/exp OR “skeletal class III malocclusion”).

In Web of Science, the search strategy was done with an advanced search using the search string as follows: (TS = Invisalign OR Clear aligner therapy OR CAT) AND TS = (lower molar distalisation OR mandibular molar distalization) AND TS = (class III malocclusion OR skeletal class III malocclusion OR dental class III malocclusion).

In Google Scholar, the search strategy was done with keywords and Boolean operators such as (“Clear aligner therapy” OR “CAT” OR “Invisalign”) AND (“lower molar distalization” OR “mandibular molar distalization”) AND (“class III malocclusion” OR “skeletal class III malocclusion” OR “Dental class III malocclusion”).

**Figure 1:**
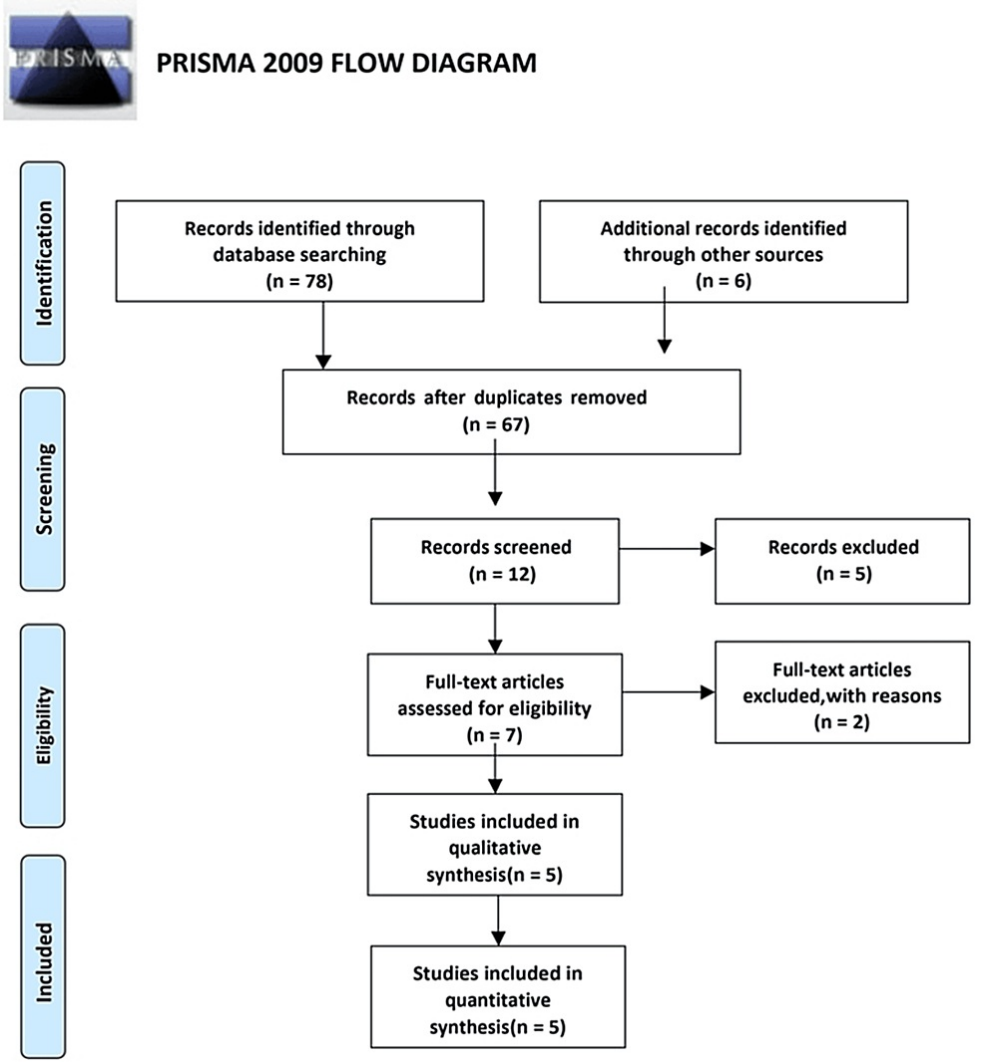
PRISMA diagram detailing the study identification and selection process. PRISMA: preferred reporting items for systematic reviews and meta-analyses

The articles were collected and segregated according to PRISMA guidelines (Figure 1). The electronic search provided numerous articles in nearly 78 articles regarding clear aligners in class III malocclusion with molar distalization. The references of the segregated articles were checked once for supplemental data.

Selection Criteria

Retrospective studies and case series showing the effectiveness of clear aligners in treating class III malocclusion with mandibular molar distalization with some predictive outcome, as well as data sets used, were the inclusion criteria for our systematic review. The study contains studies that describe the use of clear aligners to successfully distalize class III malocclusions. There were no studies on class III malocclusions that were corrected with initial teeth extracted and clear aligners. Studies that have never been published, works with only abstracts and no complete text, and documents not published in English were also disqualified. The inclusion and exclusion criteria were developed using participant, intervention, comparison, and outcome (PICO) criteria. Table 1 shows the selection criteria, outcomes obtained, and interventions in our study.

**Table 1:** Selection criteria, interventions, and outcomes obtained from our review (PICO criteria). The PICO framework focuses on the Population, Intervention, Comparison and Outcomes and it is a commonly used tool for quantitative systematic review.

Community	Class III malocclusion patients
Intervention	Clear aligner therapy
Comparison	CBCT, OPG and Lateral cephalogram
Outcome	Canine and molar relationship, Skeletal and dental changes
Study format	Case presentation and pensive analysis

Screening and Selection

The entire search and screening procedure was carried out by two examiners. The papers were chosen using inclusion and exclusion criteria based on the electronic search headings and abstracts. The first step was to eliminate unnecessary citations. The titles and abstracts were then examined to determine whether the article met our criteria. When the reviewer was certain that an article's information was unavailable, it was promptly removed from the research. In the event of a question, the complete article was downloaded for careful examination, and a second thought was provided by another reviewer. Two examiners assessed the articles collected in the first step to see whether they matched the eligibility criteria. Articles that lacked adequate design and sufficient data, as well as those that were incorrectly referenced, were eliminated. All final publications were thoroughly reviewed, and pertinent data was obtained.

Data Extraction

The first examiner retrieved data from the selected publications, which were then double-checked by another examiner. Data were acquired in the same manner from approved publications selected based on inclusion criteria. Using computer feeding (Office Excel 2013 application, Microsoft Corporation), the received data was converted to a standard electronic format. Using computer feeding (Office Excel 2013 application, Microsoft Corporation), the received data was converted to a standard electronic format. Author, year, study design, sample size, age group, intervention, treatment protocol, comparison, outcome, results, and inference were tabulated and completed in Table 2.

**Table 2:** Details of the studies that were extracted for our systematic review.

AUTHOR AND YEAR	TYPE	STUDY DESIGN	SAMPLE SIZE	AGE GROUP	STUDY GROUP	INTERVENTION	TREATMENT PROTOCOL	COMPARISON	OUTCOME	RESULTS	INFERENCE
Inchingolo et al. 2022 [12]	Invivo	Case report	1	25 years	Skeletal and dental Class III malocclusion with anterior crossbite undergoing aligner therapy	Lower molar distalization	Invisalign treatment with IPR and sequential distalization protocol of 50% of the lower arch teeth	Photographs, software staging and lateral cephalograms	Canine and molar relationships, skeletal and dental parameters	The patient reached molar and canine class I and positive overjet and overbite. The inclination of lower incisors and the interincisal angle have improved, resulting in aesthetic and functional enhancement.	sequential distalization protocol of the lower teeth leads to good compromise results in class III malocclusion
Schupp et al. 2017 [13]	Invivo	Retrospective case series	2	Case 1: 26 years Case 2: 27 years	Class III non growing patients undergoing aligner therapy	Lower molar distalization	Invisalign treatment withsequential distalisation and use of class III elastics. Use of Acceledent in case 2.	Photographs and lateral cephalograms	Canine and molar relationships, skeletal and dental parameters	Patient achieved favourable occlusion, class I relation and improvement in facial profile due to distalisation of molars	Aligners effectively correct class III malocclusion by distalisation with or without TADs. The use of Acceledent reduces the aligner wear from 7 to 5 days.
Malekian et al. 2019 [14]	Invivo	Retrospective case series	2	Case 1: 31 years Case 2: 23 years	Class III non growing patients undergoing aligner therapy with TAD or class III elastics	Lower molar distalization	Invisalign treatment with IPR in anterior mandible teeth, sequential distalisation and use of class III elastics	Photographs and lateral cephalograms	Canine and molar relationships, skeletal and dental parameters	A sequential distalization of 3 mm for case 1 and 2.5 mm for case 2 were obtained	Invisalign is effective in correction of class iii malocclusion by distalization of 2-3 mm of mandibular molars
Han et al. 2021 [15]	Invivo	Retrospective	32	24.8+2.4 years	Class III patients undergoing aligner therapy with TAD or class III elastics	Lower molar distalization	Mandibular molars distalization with clear aligner via cone beam CT (CBCT) and Dolphin software.	voxel‑based superimposition method using CBCT, OPG, lateral cephalograms	Mandibular molar distalisation of crown and root using 2D line and clipping slice functions	For mandibular first molar distalization, the average efficiency of the crown was 67.19% 9swn. 13%, and that of the root was 37.87% 7stn. 72%. For mandibular second molars, the average efficiency of the crown was 58.47% 7swn. 07%, and that of the root was 57.03% 3stn. 48%.	Clear aligners are effective in mandibular molar distalization and the movement pattern is mainly a tipping movement. To accomplish bodily movement, overcorrection should be needed.
Rota et al. 2022 [16]	Invivo	Retrospective	16	25.6 years	Class III patients undergoing aligner therapy without miniscrews	Lower molar distalization	No attachments, Sequential lower molar and premolar distalization with intermaxillary class III elastics	Pre and post treatment cephalograms	Skeletal and dental changes(6 skeletal and 15 dental variables)	Lower 2nd molar moved distally 2.47 mm, but there was a significant tipping. The first molar moved distally 1.16 mm and a significant tipping was seen. No significant skeletal changes	Invisalign is effective in moving lower molars distally by gaining tipping movement rather than bodily movement.

Evaluation of the Risk of Bias

As this review encompasses retrospective and case series data, bias risk assessment was done using the RoBANS technique. It stands for Non-randomized Risk of Bias Assessment Tool. It does not compute scores but can be used for observational study systematic reviews. It included six categories of study methodology, including participant selection, confounding variables, exposure measurement, blinding of result evaluations, incomplete data, and selective outcomes were reported. Based on this, four of our studies had a scarce risk of bias, despite one having a high risk [12].

Search and Selection of Results

The PRISMA flowchart was used to select the articles for this review. The literature search yielded 78 studies relevant to the issue, as well as another research [6]. Sixty-seven articles were eliminated because they were irrelevant, duplicated, or lacked data. After the screening step, roughly 12 articles were collected. From this, seven full-text papers were evaluated for eligibility criteria, with two articles being rejected with justification and five articles being elaborated in the current systematic review (Figure 1).

Results and discussion

Study Characteristics

This review included 5 publications that display the efficacy of clear aligners while treating class III malocclusion along with lower molar distalization. The first study was a case series given by Schupp et al. in which he found that the patients with class III achieved favorable occlusion, class I relation, and improved facial profile due to molar distalization when treated with clear aligner therapy [13]. This was followed by another retrospective case series given by Malekian et al. in which they found that Invisalign is effective in correcting class III malocclusion by molar distalization of about 2 to 3 mm [14]. This was followed by a retrospective study given by Wu et al. in which they found clear aligners are effective in class III correction with molar distalization mainly by tipping movement. He also added that overcorrection helps in achieving bodily movement of molars [15]. A case report proved that the sequential distalization protocol of lower molars helps in achieving good compromise results in correcting class III malocclusion [12]. Rota et al. published a retrospective analysis finding that the Invisalign treatment helps move lower molars distally mostly through tipping movement rather than physical movement [16].

Discussion

Class III malocclusions might have skeletal or dental etiology. In a class III skeletal discrepancy, the maxilla can be positioned backward, the mandible can be positioned forward, or both. The ANB angle is reduced to 0 degrees or less in these circumstances and the Wits analysis is negative. Non-extraction treatment with clear aligners with mandibular interproximal reduction (IPR) in conjunction with class III elastics and mandibular molar distalization can be done in non-growing patients with dental class III malocclusion or very moderate skeletal discrepancy.

The first study done based on the efficacy of treating class III malocclusion along with molar distalization was given by Schupp et al. [13]. In 2010, he presented several class II cases successfully treated with the Invisalign system with molar distalization. In 2017, he followed a similar protocol for class III malocclusion treated with lower molar distalization by presenting two cases. The first case was mild class III malocclusion in which he used Invisalign treatment only by changing the aligners once a week. The second scenario involved the non-operative treatment of a severe class III skeletal deformity employing aligners with temporary anchorage guidance and AcceleDent to provide more effective and faster mandibular molar distalization. This study discovered that using aligners to perform non-extraction and non-surgical therapy with mandibular distalization of 4mm with and without temporary anchorage devices led to favorable occlusion in class III patients. According to the first patient's case, changing aligners every seven days can cut treatment time in half. Furthermore, the adoption of an expedited orthodontic device allowed the second patient to have a five-day change of aligner wear.

Malekian et al. [14] reported a retrospective case series in 2019 describing the usage of clear aligners to achieve mandibular molar distalization in class III molar patients. He presented two cases with patients aged 31 and 23 years old with class III molar and canine relationship, non-growing patients. The treatment plan included clear aligner therapy with sequential mandibular molar distalization with class III elastics for about 22 hours per day. The amount of distal movement of mandibular molars was measured which was about 3 mm for case 1 and 2.5 mm for case 2. This was comparable with several studies done by Ravera et al. [17], Rossini et al. [18], and Simon et al. [19] which showed similar movement of molars in the maxillary arch in class II malocclusion. This study proved that Invisalign was effective in treating class III by distalizing 2-3 mm of the molar in non-growing patients with a high level of compliance achieved.

Wu et al. [15] performed a study to analyze the effectiveness of Clear aligner therapy for distalize lower molars and to guide in the clinical scenarios. In this study, 32 patients undergoing clear aligner orthodontic treatment were selected and checked for the amount of molar distalization before and after treatment. CBCT was used to measure the amount of distalization and to compare it with expected tooth movement. The amount of distalization by crown tipping was about 67.19% and the root tipping was about 37.87%. The amount of distalization of the second molar by crown tipping was about 58.47% and that of root tipping was about 57.03%. The achieved tooth movement and the expected tooth movement were statistically significant for both molars. The expected tooth movement was negatively correlated with the efficiency of the tooth crown. This showed that the Invisalign is successful in mandibular molar distalization which was majorly by tipping. Overcorrection should be done to achieve bodily movement.

Rota et al. [16] conducted a study to assess the ability of clear aligners in adults in distalizing the lower molars without mini screws. In this case study, 16 patients with a mean age of 25.6 years who had lower molar distalization with clear aligners were chosen, and lateral cephalograms were taken from them. Cephalograms were taken at the start (T0) and end (T1) of treatment. Differential skeletal and dental features were investigated. According to this study, the mandibular second molar is moved distally by 2.47 mm; however, the movement was primarily caused by tipping (p = 0.027). The first molar also showed distal movement of about 1.16 mm, which occurred predominantly by tipping (p = 0.003). The sagittal and vertical characteristics did not differ significantly. This study concluded that clear aligners are effective in lower molar distalization mainly employing tipping rather than a bodily movement which is sufficient to achieve proper molar relationship and correct basic malocclusion.

A case report was conducted which described a 25-year adult patient, presenting alongside dental and skeletal class III with a reversed overjet, along with incisal wear and tear due to traumatic occlusion came for orthodontic treatment [12]. The patient was explained with various treatment strategies, and he was willing for clear aligner therapy. A sequential distalization plan was used to correct the dental problems which were similar to the distalization protocol used for class II malocclusion in the maxillary arch. Lower molar distalization is quite difficult because of thicker bone density. Furthermore, the third molars must be extracted before proceeding with distalization. The distalizing force was achieved by utilizing class III elastics extending from the upper molar toward the lower canine, which also aids in the management of incisor proclination. The patient's canine and molar relationships were in correct class I, as well as a positive overjet and overbite. The lower anterior inclination showed good improvement with a normalized interincisal angle which provided a favorable aesthetic and functional response.

## Conclusions

According to the current evidence in this review, clear aligners were effective in treating patients with class III malocclusion in accordance with distalization of the molar. The quantity of molar distalization is around 2 to 3 mm, which aids in achieving class I canine and molar relationship, high level of compliance, and facial profile enhancement. According to the most recent findings, molar distalization gains space primarily through tipping rather than muscular movement.
